# Microstructural and Very High Cycle Fatigue (VHCF) Behavior of Ti6Al4V—A Comparative Study

**DOI:** 10.3390/ma13081948

**Published:** 2020-04-21

**Authors:** T R Jebieshia, Jong Min Kim, Jung Woo Kang, Seok Woo Son, Heuy Dong Kim

**Affiliations:** 1 Fluid Machinery Technology and Research Center (FMTRC), Daejoo Machinery, Daegu 43024, Korea; 2 Department of Mechanical Engineering, Andong National University, Andong 36729, Korea

**Keywords:** Ti6Al4V, ultrasonic fatigue test, microstructure, heat treatment, microhardness, S-N curve, VHCF

## Abstract

In this study, an investigation is carried out to evaluate and compare the material and physical properties of Grade 5 Titanium alloy (Ti6Al4V G5) samples of three different impeller manufacturers. The study aims to identify the efficient impeller core material from different Ti6Al4V G5 manufacturers. Ultrasonic fatigue test for Ti6Al4V samples of 100 horsepower (hp) centrifugal compressor impeller parts is performed before and after heat treatment. The effect of microstructure on Very High Cycle Fatigue (VHCF) behavior of Ti6Al4V is also analyzed and discussed in detail. Optical Microscopy (OM) and Scanning Electron Microscopy (SEM) observation are carried out to investigate the microstructure of different Ti6Al4V material samples. The dynamic elastic properties are measured by the Impulse Excitation Technique (IET) at room temperature. The fracture behavior of the tensile specimens is analyzed by SEM. Post-heat-treatment analysis of Ti6Al4V is also carried out and presented which affects the grain size of the material sample and thus considerable effect in the mechanical properties. Chemical composition investigation of Ti6Al4V using SEM and Energy Dispersive X-ray Spectroscopy (EDS) also included in this study.

## 1. Introduction

The demand for Titanium alloy (Ti6Al4V) dominates in various fields including aerospace, marine, automobile, chemical, and biomedical industries [1,2,3,4,5,6]. This widespread use of Grade 5 Ti6Al4V is as it possesses high specific strength, high fracture toughness, ductility, excellent corrosion, and fatigue resistance, high-level biocompatibility, good damage tolerance, workability, etc. [1,7,8,9,10,11]. This lightweight yet strong alloy saves weight in highly loaded structures and hence is extremely suitable for gas turbines and many airframe components as well as for automotive components and biomedical devices [12,13]. The characteristics of each titanium alloy grade are different and used for various purposes. Typical titanium alloys include Grade 5 (Ti6Al4V or TC4), Grade 7 (Ti0.15Pd), Grade 12 (Ti0.3Mo0.8Ni), Grade 23 (Ti6Al4V Eli), etc. Grade 5 Ti6Al4V is well suited for applications such as compressor blades, discs, and rings for jet engines; pressure vessels; rocket engine cases; helicopter rotor hubs, etc. Grade 5 titanium alloy is fully heat treatable (solution heat treatment and aging) to increase strength and has good welding and fabrication characteristics. Grade 1 to Grade 4 poses tensile strength of 240–740 MPa whereas that of Grade 5 is from 900 to 1200 MPa. Leyens et al. [7] explained the properties and applications of different grades of titanium alloys in detail. Williams et al. [14] elaborated on the use of Ti6Al4V in the aircraft industry. As the industry advances, there is a need for efficient research to ensure the durability and the reliability of the material along with the life prediction. As the life expectancy increases, the core components sustaining extremely high numbers of cycles of loads will face increasingly severe challenges in gigacycle regions and it is necessary to study the very high cycle fatigue (VHCF) or giga cycle fatigue (GCF) performance. Various studies [15,16] are available on VHCF (over $10^{7}$ cycles) and GCF (above $10^{9}$ cycles). The VHCF and GCF tests were carried out in the range from $10^{7}$ to $10^{10}$ on ultrasonic fatigue testing machine at the frequency of 20 kHz. TC-17, TC-21, Ti-6Al-7Nb, Ti-22V-4Al are some of the important titanium alloys for which the VHCF and GCF behavior has been investigated in the past [17]. In the advanced academic world, many applications are being thoroughly pursued through the ASTM as well as the Giga cycle stop test method as a standard. Marines et al. [18] suggested that S-N curves must be generated to guarantee the real fatigue strength in the high cycle regime as those have a great impact on the design field. Recent studies [18,19] using ultrasonic test systems have shown that many materials including some steels and titanium alloys exhibit a sharp decrease in fatigue strength between very high fatigue lives of $10^{6}$ and $10^{9}$ cycles, which is in contrast to the classical concept of fatigue limit.

Ti6A14V is an α−β alloy in which the α-stabilizer is enriched aluminum, and the β-stabilizer is enriched vanadium [4]. The response of this titanium alloy to heat treatment are discussed in terms of the phase relationships. Ti6Al4V materials are generally cooled by annealing until the solute atoms are completely solid in a single phase by annealing until the β-phase is completely dissolved to prevent diffusion. In this state, the alloy has relatively soft and weak properties. Thus, the α−β phase can coexist through secondary or precipitation heat treatment to form β-precipitated phase in the form of finely dispersed particles which enhances the mechanical properties. After heat treatment, the alloy achieve superior tensile thermo-mechanical behavior. Several authors [6,20,21,22,23,24] discussed the effect of various heat-treatment procedures on the microstructure, hardness and tensile properties of Ti6Al4V. Gao et al. [25] concluded that the annealing treatment decreases the fatigue life of Ti6Al4V. Zuo et al. [2] carried out the fatigue tests of Ti6Al4V microstructures and deduced that there is a dependence of the fatigue life on the location and size of the crack initiation behavior.

The aim is to identify the efficient core component materials for 100 hp centrifugal compressor impeller by analyzing the characteristics relatively and comparing the data of different impeller manufacturers. To identify the best material quality specimen, an attempt is made to evaluate the basic properties by tensile strength test and dynamic elasticity and Poisson’s ratio by using Impulse Excitation Technique (IET) method. This paper also analyzes the microstructure and hardness of Ti6Al4V before and after heat treatment and summarizes the findings and compares the tensile properties and fatigue performance of Ti6Al4V from different manufacturers. Also, fatigue fracture of the surface is observed with the help stress-versus-cycle fatigue cracks graphs.

## 2. Experimental Procedures

**Figure 1:**
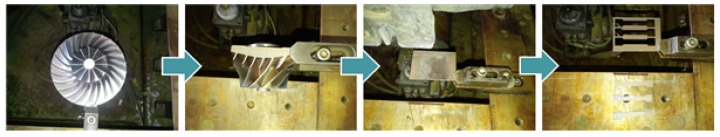
Wire-cutting process for test samples from the impeller material.

**Figure 2:**
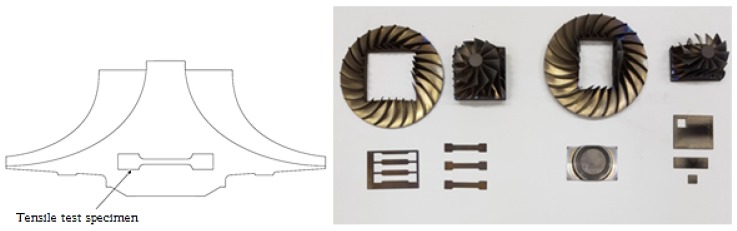
Impeller test specimens.

Titanium alloy (Ti6Al4V) samples from three different manufacturers are considered and named A, B, and C, in which the material sample A is also considered for the heat-treatment study. The Wire Cutting Electrical Discharge Machine (WCEDM) method in Figure 1 is the most precise and standard process used to machine the specimens. Figure 2 shows the bulk volume of the impeller and the specimens for various testing and machined from the lower center part of the impeller. The center part of the impeller part is first secured with a rectangular plate, and the ductility, tensile specimen, microhardness, and specimen are prepared for testing.

**Figure 3:**
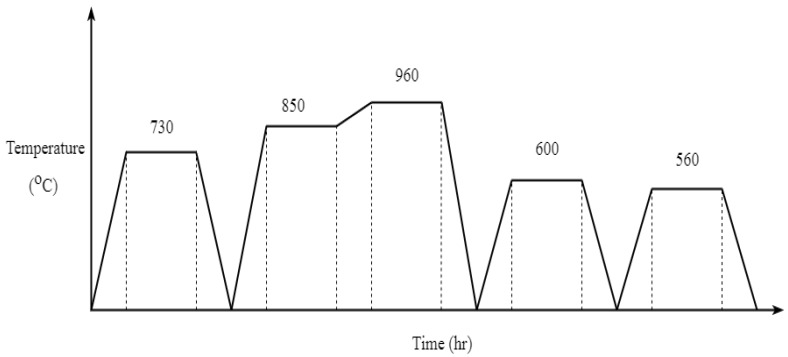
Heat-treatment process for material sample A.

The heat treatment of the Ti6Al4V material allows the solute atoms to be completely solid, which are cooled after holding until the β phase is completely dissolved to prevent diffusion and subsequent formation of the β phase. The relevant basic histological details of the Ti6Al4V alloy with the phase state diagram and the basic microstructure are explained by Leyens et al. [7]. The microstructure and mechanical property change are observed to occur according to the heat-treatment conditions. Figure 3 shows the heat-treatment profile details of material sample A used for this study. Commonly used heat-treatment methods for Ti6Al4V are annealing, solution heat treatment, aging treatment and stress relief annealing. Annealing is to achieve better overall properties by eliminating internal stress and improving plasticity and tissue stability. Generally, the annealing temperature of the alloy and (α−β) alloy is 120–200 ${}^{\circ }$C lower than the (α−β) beta phase transition temperature. Solid solution heat treatment is rapidly quenched in a high-temperature region to obtain a martensite alpha phase and a semi-stable beta phase and then decomposed by maintaining these semi-stable phases in an intermediate temperature region to finely disperse a second phase such as an alpha phase or a compound.

The heat treatment is conducted in the following steps:

1. Heating to 730 ${}^{\circ }$C and holding for 2 h; air-cooling to room temperature (annealing)
2. Heating to 850 ${}^{\circ }$C and hold it for 2 h and reheat to 960 ${}^{\circ }$C and holding it for 2 h; water-cooling to room temperature (solution heat treatment)
3. Heating to 600 ${}^{\circ }$C (aging treatment) and 560 ${}^{\circ }$C (stress relief annealing) at each temperature holding for two hours and air-cooling to room temperature

### 2.1. Dynamic Modulus of Elasticity and Poisson’s Ratio Measurement by Impulse Excitation

**Figure 4:**
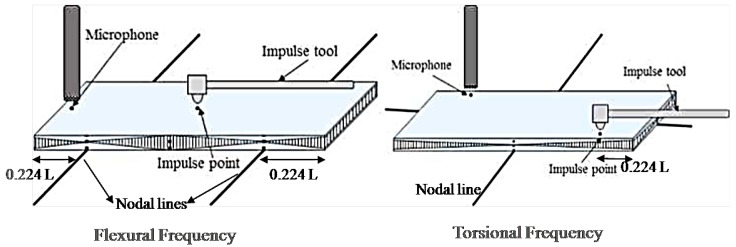
Schematic diagram of Impulse Excitation Technique (IET).

The Impulse Excitation Technique (IET) measurement is experimentally carried out based on the standard ASTM E1876-01 [26]. IET measures the fundamental resonant frequency of test specimens by exciting them mechanically with an impulse tool. Specimen supports, impulse locations, and signal pick-up points are selected to induce and measure specific modes of the transient vibrations. A transducer senses the resulting mechanical vibrations of the specimen and transforms them into electric signals [27]. The appropriate fundamental resonant frequencies, dimensions, and mass of the specimen are used to calculate dynamic Young’s modulus, dynamic shear modulus, and Poisson’s ratio. In this testing, rectangular specimens of length 60 mm, width 20 mm and thickness 3 mm are prepared and placed on the node line to accurately determine the natural frequency and transverse waves of the material. The specimens are excited using the impulse tool shown in the schematic of IET, Figure 4, and the signal is received and analyzed through a non-contacting microphone to measure the fundamental flexural frequency and the torsional frequency. In the case of flexural frequency, the nodal lines where the specimens are supported at about 0.224 L from both ends of the specimen and the microphone is placed at the end of the specimen. The torsional frequency is generated by placing the nodal line at the midpoint of the length and width of the sample (Figure 4). The microphone is placed at 0.224 L from one end of the sample.

**Figure 5:**
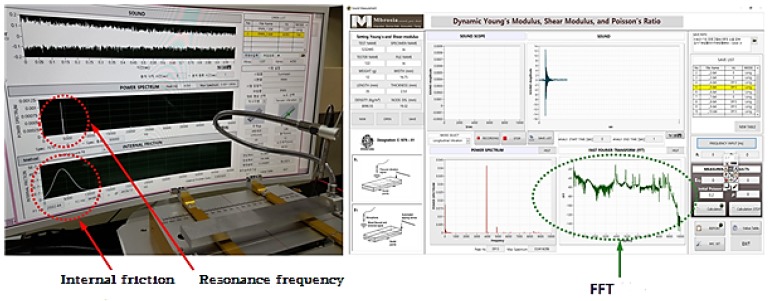
Setup for dynamic Young’s modulus measurement.

As in the program shown in Figure 5, each sound frequency is derived by Fast Fourier Transform (FFT) analysis and then the substituted into the relational expressions, to calculate the dynamic elastic modulus, the shear coefficient and the Poisson’s ratio. The dynamic elastic modulus and Poisson’s ratio will be useful to derive the damping coefficient using the resonance frequency peak of the material in the future due to the characteristics of the impeller environment.

### 2.2. Micro Hardness

Vickers microhardness test is carried out to identify the resistance of the material to indentation such as permanent deformation. The ASTM specimens of Ti6Al4V material loaded at 0.2 mm/s under load control. The critical cross-sections of the specimens are diamond polished (mirror finishing) to the final condition. The microhardness measurement test was averaged over five measurements under a maximum load of 500 g with a Vickers diamond tool indenter to obtain the Vickers Pyramid Number (HV).

### 2.3. Tensile Test

**Figure 6:**
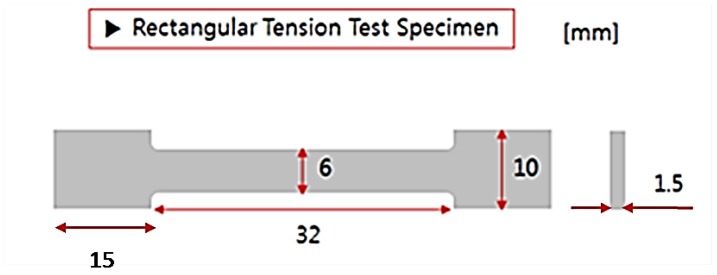
Details of ASTM E8 tensile test specimen.

Figure 6 shows the ASTM test sub-size specimen standard details where the specimens are machined from the middle of the impeller. All specimen dimensions (flat bar) and test conditions are following ASTM E8 on an Instron Load Frame at room temperature. All tensile tests were conducted at a constant strain rate of 0.005 mm/min. In each test, the yield and ultimate strengths were recorded in addition to the elongation to failure.

### 2.4. Microstructural Observation

The microstructure of an alloy is an important factor that determines its hardness, tensile properties, fracture toughness, fatigue resistance and resultant fracture behavior [28]. The microstructure characteristics of the original and heat-treated Ti6Al4V alloy specimens were characterized by Zeiss Optical microscopy (OM) and Scanning Electron Microscopy (SEM). To observe the microstructure, a solution of 92% distilled water, 6% nitric acid and 2% hydrofluoric acid is used as the etching solution. The chemical composition details of different Ti6Al4V material samples also analyzed and presented.

### 2.5. Ultrasonic Fatigue Test

**Figure 7:**
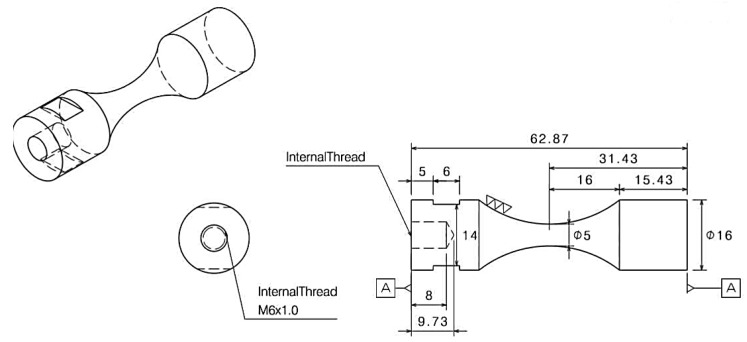
Specification of ASTM E8 ultrasonic fatigue test specimen.

**Figure 8:**
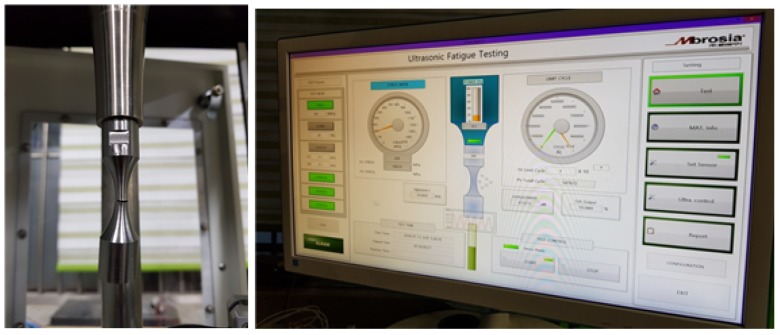
Ultrasonic fatigue test setup.

Ultrasonic fatigue technique is used to determine the fatigue behavior of Titanium alloy in a range of $10^{6}$ to $10^{9}$ cycles under fully reverse loading and in room temperature. Fractographic observations were performed using standard SEM (JEOL-6510) of JEOL Ltd., Japan, to analyze the representative fracture surfaces. The generated data on the fatigue behavior of Ti6Al4V are related to high cycle fatigue with the use of a stress-life approach. Fatigue specimens were machined from Ti6Al4V Grade 5 round bar with 12.7 mm diameter to a reduced uniform gauge section (Figure 7). Figure 8 shows the hourglass-shaped test specimen, which was designed based on the dynamic modulus values of the material samples A, B, and C. Table 1 shows the test conditions for the ultrasonic fatigue test. A displacement-controlled fatigue test method using 20 kHz Piezoelectric Ceramic (PZT) shotgun vibration is carried out with the stress amplitude range of 450–750 MPa at room temperature. The ultrasonic fatigue tests are conducted under a fully reversed loading condition, minimum to maximum load, i.e., stress ratio, R = −1. The feedback function of the displacement sensor in Figure 8 is set so that the constant strain is maintained while the test piece is fastened. To prevent deterioration, air-cooling is concentrated in the center part of the specimen where the maximum stress is applied. When the fatigue fracture occurred during the test, the ultrasonic resonance was released and the cycle was automatically stopped, and the cycle is recorded and thus the fatigue life according to each stress amplitude can be estimated.

## 3. Results and Discussion

In this section, the results obtained from the experimental tests explained in Section 2 are presented and discussed in detail.

### 3.1. Dynamic Elastic Modulus and Poisson’s Ratio

The dynamic Young’s modulus, shear modulus and Poison’s ratio for material samples A, A-H, B, and C obtained from the tests are listed in Table 2. The results summarize the average material properties after repeated measurements in five specimens for each material samples and the values are within the acceptable range, 100–120 GPa [29]. Table 2 reveals that the material sample C poses the highest Young’s modulus, 114 GPa and the material sample B poses the lowest, 105 GPa, as compared with that of the other Ti6Al4V material samples. A maximum difference of about 9 GPa is found between the different impeller manufacturers. And also, it is clear that for the material sample A before and after the heat treatment are the same with the difference in elastic and shear modulus values is of about 1 GPa. According to the supplier data of material sample C, Young’s modulus of elasticity and shear modulus should be 113.763 GPa and 43.988 GPa respectively and the variation of present results with the standard values are 0.26% and 0.35% respectively. For all the material samples the ideal density value is 4430 kg/m${}^{3}$. From the current results, the error identified in the density values for material samples A, A-H, B, and C is 1.22%, 1.81%, 1.04%, and 0.79% respectively. The difference in density of present results and standard value is higher (1.81%) for the heat-treated material sample (A-H).

### 3.2. Microhardness

**Figure 9:**
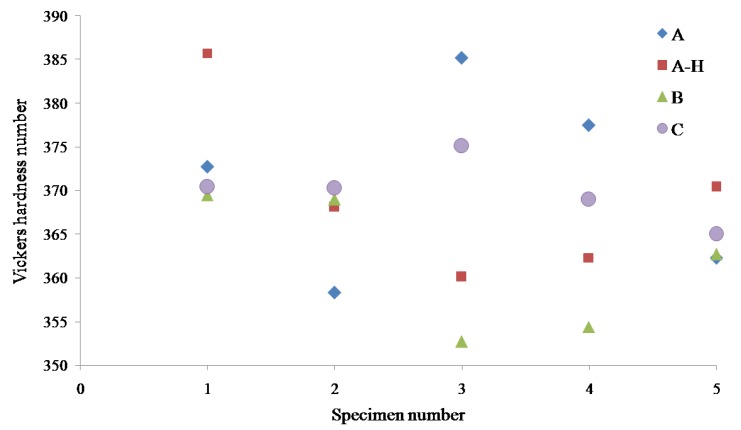
Hardness test results of Ti6Al4V material samples.

The microhardness test was carried out by measuring the Vickers hardness pyramid number (HV). Figure 9 shows the results of microhardness measurement for all the four Ti6Al4V material samples, and the material sample B is about 10 units lower than the other two samples. The standard deviations of the hardness values of material samples A, A-H, B, and C illustrated in Figure 9 are measured as 10.99, 10.06, 7.89 and 3.61, respectively. The standard deviation values of all the samples are much lesser than the mean which explains the data is much clustered around the average hardness values. The material sample C poses the smallest relative standard deviation, 0.97%. It is also found that there is no considerable difference in the average hardness value before and after the heat treatment of material sample A (Table 3). The standard Vickers hardness number for material sample A is 376 according to the supplier datasheet and the variation with current experimental average data is 1.28%. The Vickers hardness pyramid number of all the specimens of material samples A, A-H, B and C are within the acceptable limits, 300–400 [7].

### 3.3. Tensile Test

**Figure 10:**
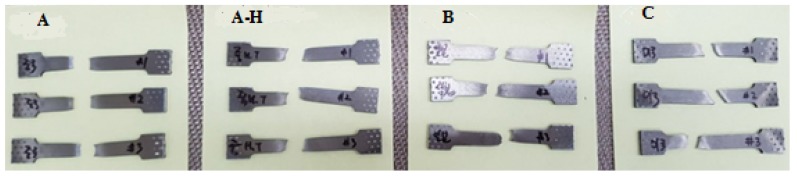
Tensile specimens of different Ti6Al4V samples (A, A-H, B and C) after the tensile test.

Figure 10 shows the tensile test samples after the experiments mentioned in Section 2.3. Table 4 shows tensile test results for three Ti6Al4V materials, respectively, and it is evident that all the material samples A, B, and C poses almost the same maximum tensile strength values. It is also found that the elongation of the material sample C is lower compared to that of material samples A and B. The standard range of elongation at break for all the material samples is from 10–18% in accordance with the datasheet. The maximum tensile strength of all the material samples are fitted within the acceptable span, 900–1200 MPa [7]. The tensile strength of material sample B and C must be greater than 895 MPa and for the material sample A, the tensile strength must be within the range of 895–1103 MPa according to the supplier datasheet. For all the specimens, the tensile strength values are within the permissible limit as recorded in the technical datasheet from the supplier.

### 3.4. Microstructure Investigation and Chemical Composition

**Figure 11:**
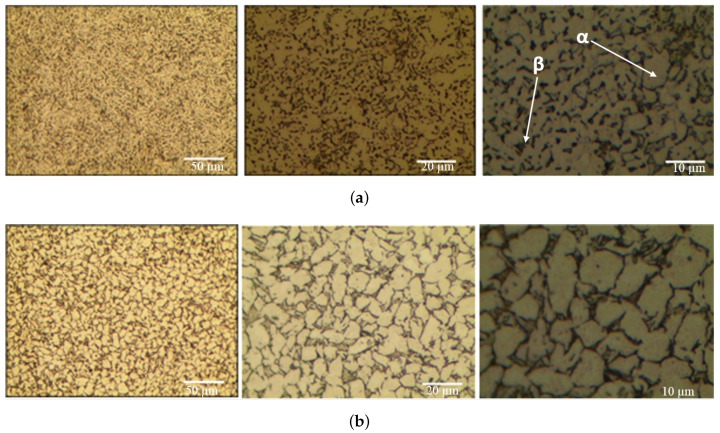

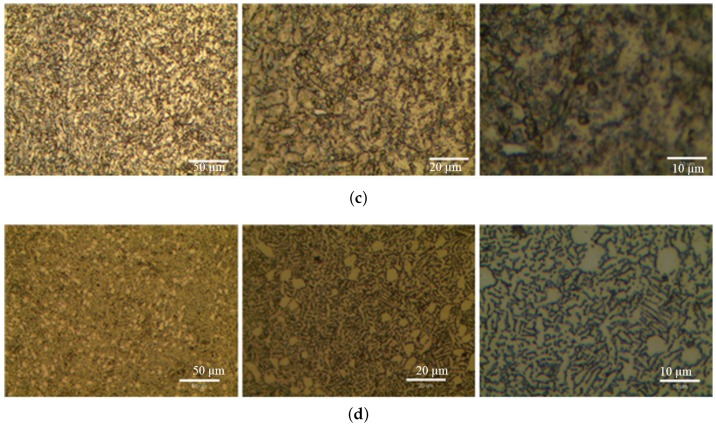
Ti6Al4V microstructure–Optical microscopy (OM). (**a**) Material sample A; (**b**) Material sample A-H; (**c**) Material sample B; (**d**) Material sample C.

**Figure 12:**
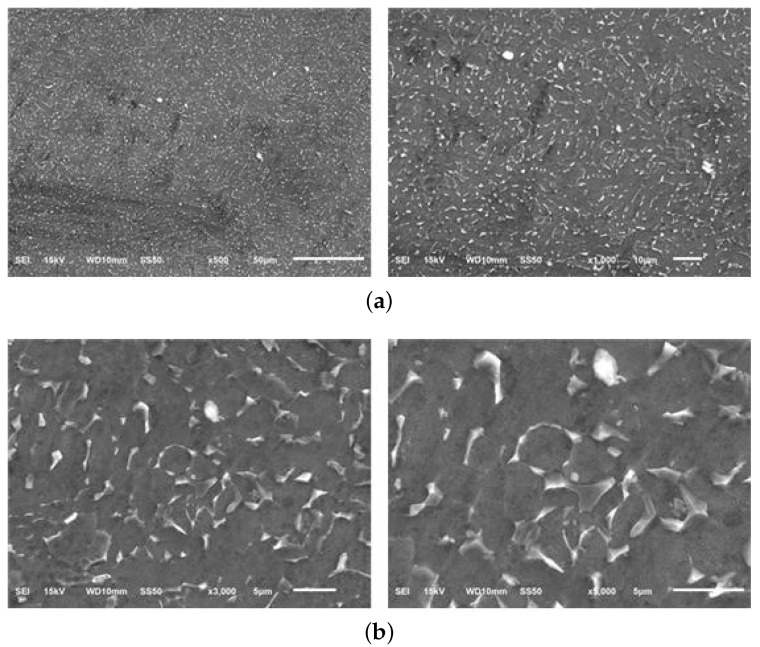
Microstructure of Material sample A–Scanning Electron microscopy (SEM). (**a**) Material sample A (500 and 1000×); (**b**) Material sample A (3000 and 5000×).

**Figure 13:**
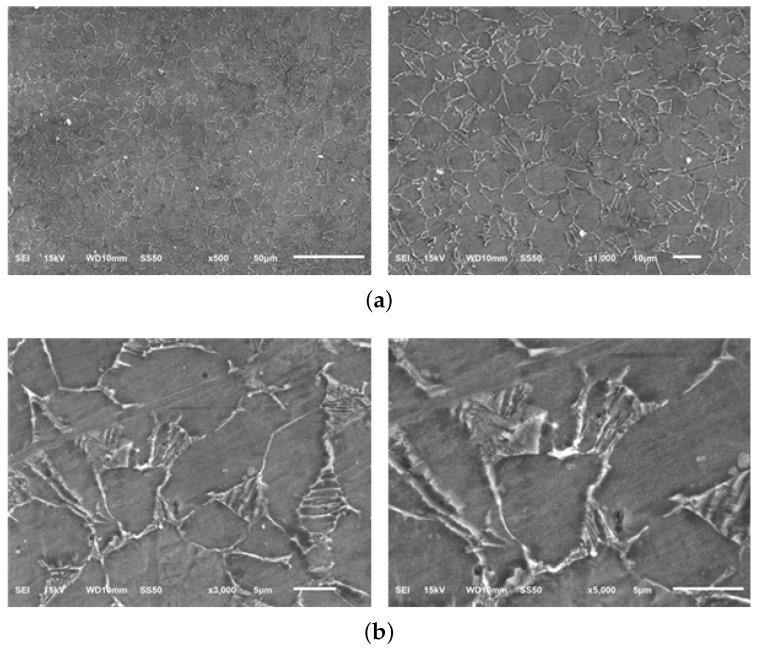
Microstructure of Material sample A-H—Scanning Electron microscopy (SEM). (**a**) Material sample A-H (500 and 1000×); (**b**) Material sample A-H (3000 and 5000×).

**Figure 14:**
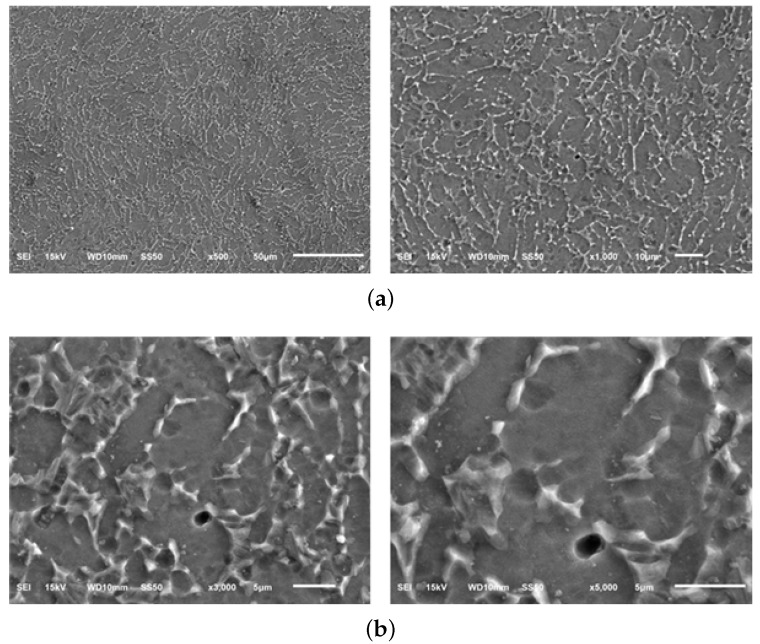
Microstructure of Material sample B—Scanning Electron microscopy (SEM). (**a**) Material sample B (500 and 1000×); (**b**) Material sample B (3000 and 5000×).

**Figure 15:**
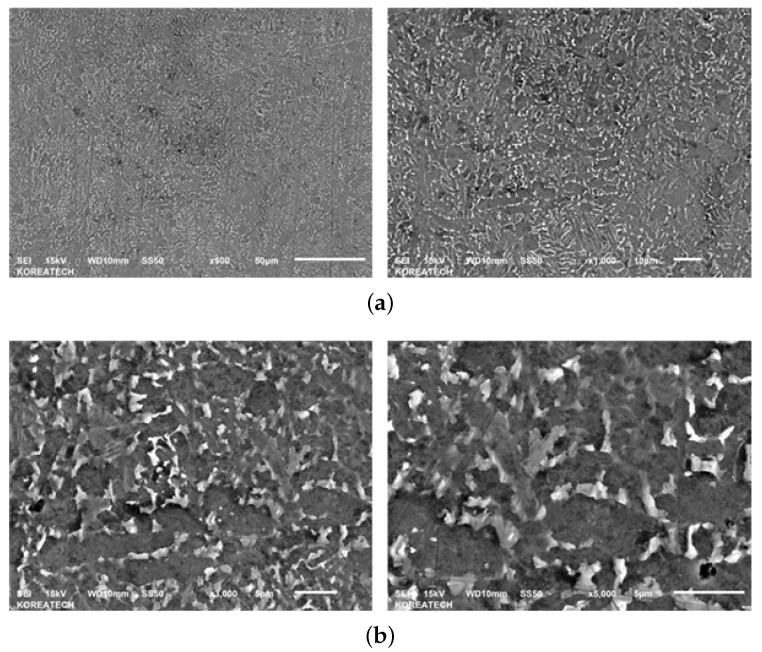
Microstructure of Material sample C—Scanning Electron microscopy (SEM). (**a**) Material sample C (500 and 1000×); (**b**) Material sample C (3000 and 5000×).

The micrographs of material samples A, A-H, B, and C, for 50, 200, 1000 times magnification from the Optical microstructural investigation is shown in Figure 11. As shown in the figure, the microstructures of different material manufacturers are slightly different and in material sample C, coarse grains of about 5 μm are identified. Figure 12, Figure 13, Figure 14 and Figure 15 shows the microstructural details of different Ti6Al4V alloy material samples with 500, 1000, 3000 and 5000 times magnification using SEM. The rounded tissue is the alpha phase and the black part that appears stratified is the beta tissue. Typical Ti6Al4V alloy structure with heat treatment is arranged in an α phase structure with a long layer structure. The β phase transformed into the Ti6Al4V alloy structure after the heat treatment is formed into the middle intermediate lamella shape between the α phase grains, so that α phase and α+β phase coexist. The α phase and the α+β phase are observed before and after the heat treatment using a precision optical microscope and an SEM.

**Figure 16:**
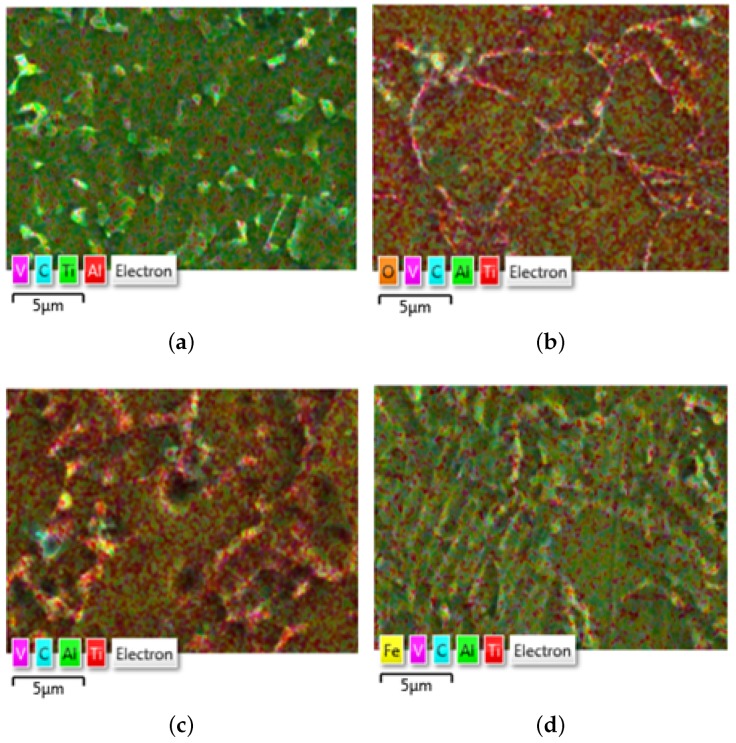
EDS layered image. (**a**) Material sample A; (**b**) Material sample A-H; (**c**) Material sample B; (**d**) Material sample C.

**Figure 17:**
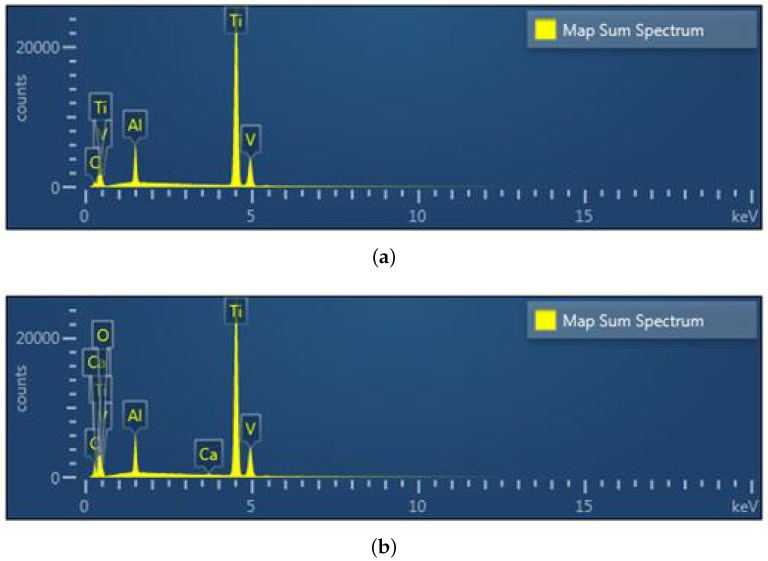

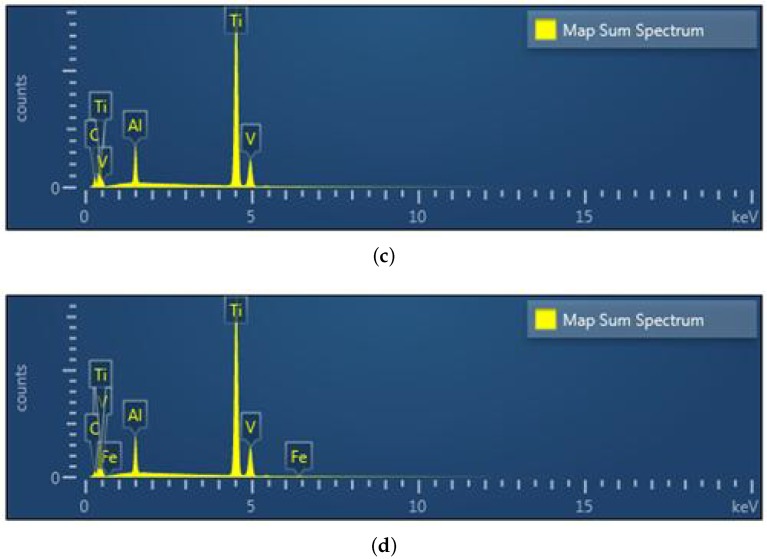
EDS spectrum analysis of the material samples. (**a**) Material sample A; (**b**) Material sample A-H; (**c**) Material sample B; (**d**) Material sample C.

The chemical composition (weight and atomic in %) of Ti-6Al-4V obtained from the microstructure investigation as follows (electron Volts eV versus No. of counts per channel). An SEM with Energy Dispersive Spectroscopy (EDS) elemental chemical composition analysis identified the elements present in the Titanium alloy Figure 16 and are listed in Table 5 for material samples A, A-H, B, and C. The magnitude of the atomic weight of different inclusive elements determines the characteristics of the material. The concentration of contamination, discoloration, or deposit on a surface are identified using EDS mapping. The EDS mapping in Figure 17 shows the lateral distribution of the contamination in SUM spectrum, how well a thin coating is covering a surface or if there are scratches in a coating and it is clear that 6% of Al and 4% of V is presented in the Ti substrate. Presence of Calcium (Ca) content found in material sample A-H and Iron (Fe) content in material sample C.

### 3.5. Ultrasonic Fatigue Test and Lifetime Prediction

**Figure 18:**
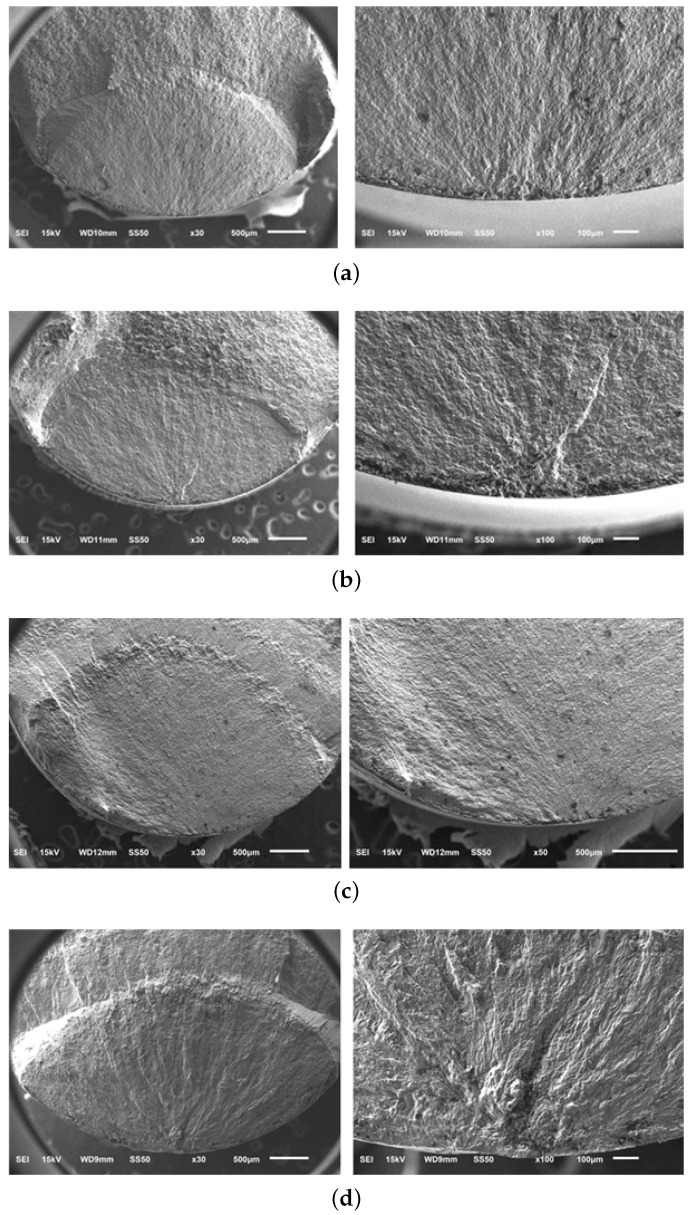
SEM micrographs of ultrasonic fatigue test. (**a**) Material sample A; (**b**) Material sample A-H; (**c**) Material sample B; (**d**) Material sample C.

**Figure 19:**
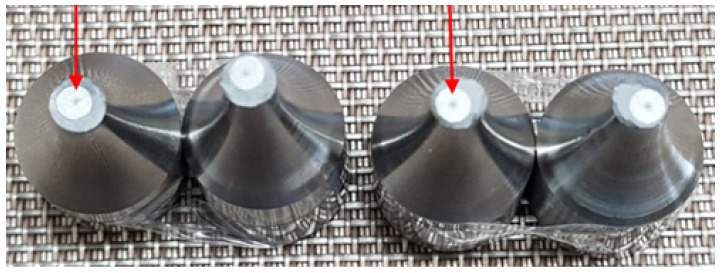
Fatigue fracture of material sample C.

**Figure 20:**
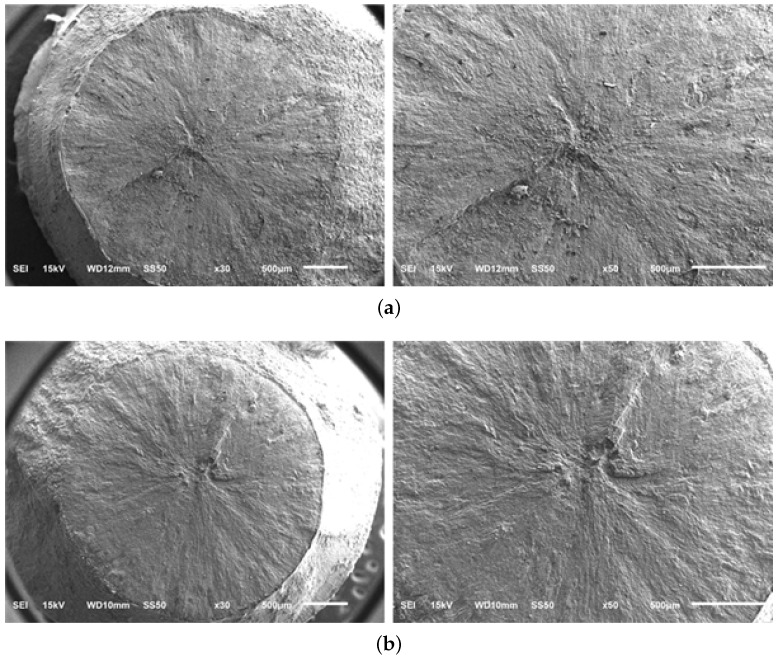
SEM micrographs of fatigue fractured surface of material sample C. (**a**) Stress amplitude 575 MPa and no. of cycles to failure 9.24 × $10^{7}$; (**b**) Stress amplitude 650 MPa and no. of cycles to failure 4.7 × $10^{6}$.

Figure 18 shows the fatigue fracture profiles of A, A-H, B, and C materials, and the starting point of the fatigue crack progressed from the surface. It is confirmed that the fatigue fracture occurred radially to the surface starting point as a result of SEM observation of the fatigue stress concentrated region by selecting the sample according to the cycle among the total fatigue fractured test specimens. However, Figure 19 shows that crack initiation point is generated inside the fatigue fracture only in material sample C; specimen # 7 (575 MPa − 9.24 × $10^{7}$) and # 14 (650 MPa − 4.7 × $10^{6}$). Figure 20 shows the results of SEM observation of specimens fractured in specimens # 7 and # 14 internal starting point of C material sample mainly due to the failure feature called “fish eye” described in [2,30]. This is because the alpha phase is uneven and the crystal grain between alpha and alpha is destroyed by stress concentration. The overall fatigue strength of the material sample C is high, but the identification of internal fatigue failure stress concentration is an important concern in high-speed parts such as impellers.

In the case of internal point breakage, stress concentration occurs between grain boundaries. In this case, several surface treatments are performed to improve fatigue life. However, when the fatigue fracture of the internal base point occurs, the surface treatment becomes useless. From the results, it is noticeable that microstructure with large grain size is observed in material sample C and it is concluded that the fatigue fracture is caused by this crystal grain as a notch in the fatigue test. Notched specimens processed larger cleavage fracture zones when compared to un-notched specimens exhibiting low ductility. At macro-scale, stress concentration arises from geometrical discontinuities and it affects strength, ductility, and fracture of alloys. At micro-scale, a different stress state and higher stress concentration can occur at grain boundaries due to the presence of impurities, dislocations pileup, etc. and can hence cause fatigue fracture [31].

**Figure 21:**
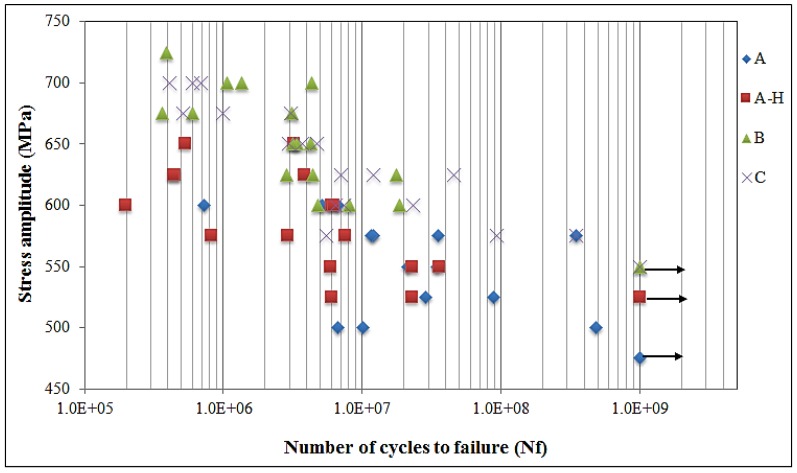
Fatigue results of different Ti6Al4V samples (arrows denote the run-out specimens).

Figure 21 shows the fatigue curve comparison data of different impeller manufacturers. Giga cycle fatigue strength of material samples B and C is 550 MPa which is slightly higher than that of material sample A (470 MPa) and A-H (530 MPa). In particular, when checking the linear distribution of the fatigue diagram, B and C material samples showed a tendency to clustered against certain stress, but in the case of material sample A, the dispersion is significantly higher. The measured standard deviations of the stress amplitude of material samples A, A-H, B, and C are 36.84, 40.82, 40.01, and 43.93 MPa, respectively. For the material sample B, the dispersion of data is lower as the coefficient of variation is smaller, 6.11%, compared to all other samples. Also, the fatigue limit in the giga cycle stop is the same for the B and C material samples, and for the material sample A, the giga cycle stop is high with the solution heat treatment. It can be concluded that in the ultrasonic fatigue test, the difference is severely seen by the same specimen size and the same stress band criteria for different impeller manufacturers.

## 4. Conclusions

In this study, the physical and mechanical data of Ti6Al4V used in impeller materials are evaluated and compared. In addition to the comparative study on different impeller manufacturers, an attempt is made to assess the material and physical characteristics before and after the solution heat treatment of the test specimens. The mechanical properties of the present experimental results are compared with that of the supplier datasheet and with the available literature. The microstructure and fatigue performance of different Ti6Al4V alloys are investigated and presented in detail. In the case of material sample C, there were a few non-metallic inclusions, and fatigue cracks in titanium are found at the internal starting point, which is used as important data for quality evaluation and reliability evaluation of impeller in very high cycle fatigue test according to microstructure. It is difficult to classify the fatigue test results as the overall fatigue life is evaluated as superior for all the material samples. The major conclusions derived from this study are:

- The dynamic elastic modulus and Poisson’s ratio were measured using the IET. From the results, it is evident that the material sample from manufacturer C poses the highest 114 GPa and the B material sample poses the lowest at 105 GPa which is within the standard permissible limit.
- Measurement of the microhardness analysis showed that the Vickers hardness values (HV) for A, A-H, B, and C material samples are between 360 and 370. The microhardness analysis showed that before and after the heat treatment is almost the same with 10 unit difference (0.51%).
- Using the process of microstructure investigation, the different raw materials present in each material model are estimated using the EDS mapping process. As a result of element composition mapping, Ti6Al4V basic components could be confirmed as a whole, but trace impurities of Ca were detected in the heat-treatment material sample A and trace of Fe in material sample C.
- From the tensile test, it can be seen that the maximum tensile strength value is almost the same for all the material samples, but it is also found that the material sample C is lower than that of materials of A and B at the elongation. Tensile test results before and after the heat treatments, the material sample A possesses the lowest. However, this error might be due to the sub-size plate specimen in the experiment.
- The fatigue fracture of each stress band cycle is observed, but most of the surface-based fatigue failure occurred, but in the case of material sample C, it is found that the fatigue fracture occurred at the internal origin due to large size crystal grain as a notch.
- For material model A, the fatigue strength is not only low but the data dispersion is observed highly scattered. But for material samples B and C are comparatively clustered. Moreover, material samples B and C possess slightly higher fatigue strength than A and A-H.
- The material and physical properties of impeller parts are considered to be the quality evaluation standard, thus the material sample C can be used as a high-level stable impeller core part. Moreover, the material properties of sample C firmly agree with that of the supplier technical datasheet compared to the other two material samples. Thus, according to the present experimental study, material sample C can be used as the efficient impeller core component.

## Figures and Tables

**Table 1:** Conditions of ultrasonic fatigue test.

Loading Frequency	Stress Ratio (R)	Stress Range	Cycle	Test Atmosphere
20 kHz	−1	450–750 MPa	10${}^{4}$–$10^{9}$	Room temperature

**Table 2:** Average properties of each material samples.

Sample	Frequency (Hz)		Young’s Modulus	Shear Modulus	Density	Poisson’s Ratio
	1	2	(GPa)	(GPa)	(kg/m${}^{3}$)	
A	4789	8714	109.547 ± 0.2	43.078 ± 0.2	4376	0.271
A-H	4752	8630	109.417 ± 0.2	42.618 ± 0.2	4350	0.283
B	4602	8318	105.149 ± 0.2	40.811 ± 0.2	4384	0.288
C	4762	8622	114.064 ± 0.2	44.143 ± 0.2	4395	0.291

**Table 3:** Vickers hardness number of Ti6Al4V.

Sample	A	A-H	B	C
1	372.7	385.7	369.5	370.4
2	358.3	368.1	369.0	370.3
3	385.2	360.1	352.7	375.1
4	377.5	362.3	354.4	369.0
5	362.3	370.4	362.7	365.0
Average	371.2	369.3	361.7	370.0

**Table 4:** Tensile test result.

Sample	A		A-H		B		C	
	#1	#2	#1	#2	#1	#2	#1	#2
Maximum tensile strength (MPa)	938	959	911	903	939	997	934	962
Elongation (%)	19.1	20.1	18.7	18.5	18.6	20.0	16.4	17.6

**Table 5:** Chemical composition of Ti6Al4V.

Material Sample	Weight (%)	Element						
		Ti	Al	V	C	O	Ca	Fe
A	Weight (%)	87.97	6.02	3.94	2.07	-	-	-
	Atomic weight (%)	79.54	9.66	3.35	7.45	-	-	-
A-H	Weight (%)	79.16	5.27	4.03	5.98	5.29	0.27	-
	Atomic weight (%)	59.83	7.08	2.86	18.03	11.96	0.24	-
B	Weight (%)	85.64	5.81	3.56	4.99	-	-	-
	Atomic weight (%)	71.85	8.66	2.80	16.68	-	-	-
C	Weight (%)	86.46	5.87	4.75	2.59	-	-	0.33
	Atomic weight (%)	77.24	9.30	3.99	9.21	-	-	0.25
